# Age-appropriate compliance and completion of up to five doses of pertussis vaccine in US children

**DOI:** 10.1080/21645515.2018.1502526

**Published:** 2018-08-29

**Authors:** Girishanthy Krishnarajah, Elisabetta Malangone-Monaco, Liisa Palmer, Ellen Riehle, Philip O. Buck

**Affiliations:** a GSK, Philadelphia, PA, USA; b Truven Health Analytics, an IBM Company, Bethesda, MD, USA; c Truven Health Analytics, an IBM company, Ann Arbor, MI, USA

**Keywords:** Pertussis, DTaP, vaccination, Medicaid, children, compliance, United States

## Abstract

**Background**: In the United States (US), diphtheria, tetanus, and acellular pertussis (DTaP) vaccination is recommended at 2, 4, and 6 months (doses 1–3), 15–18 months (dose 4), and 4–6 years (dose 5). The objective of this study (GSK study identifier: HO-14–14383) was to examine DTaP completion and compliance rates among commercially insured and Medicaid-enrolled children. Secondarily, the study aimed at identifying predictors of compliance/completion.

**Methods**: *Truven Health MarketScan* Commercial and Multi-State Medicaid databases (2005–2013) were analyzed separately. Children born during 2005–2011 with ≥ 2 years continuous enrollment from birth provided data for doses 1–4; those with continuous enrollment from birth to their seventh birthday provided dose 5 data. Series compliance (each recommended dose by 3, 5, and 7 months; 19 months; seventh birthday) and completion (3 doses by 8 months; 4 by 24 months; 5 by seventh birthday) were calculated. Predictors of compliance/completion were identified using multivariable logistic regression.

**Results**: A total of 367,493 commercially insured and 766,153 Medicaid-enrolled children were followed for ≥ 2 years; and 23,574 and 41,284, respectively, for ≥ 7 years. Series compliance to doses 1–3, 1–4, and 1–5 were 67.2%, 55.3%, 47.5% (commercial) and 37.4%, 27.3%, 14.4% (Medicaid), respectively. Predictors of better compliance/completion included: later birth year (commercial/Medicaid) and higher household income (commercial); predictors of worse compliance/completion included: Northeast residence (commercial), birth hospitalization ≥ 14 days (commercial/Medicaid), and Black race/ethnicity (Medicaid).

**Conclusions**: DTaP series compliance/completion improved over time, but appear to be suboptimal. As this could increase pertussis risk, greater awareness of the importance of timely vaccination completion is needed.

**GSK study identifier**: HO-14–14383

## Background

In the United States (US), primary immunization with diphtheria, tetanus, and acellular pertussis vaccine (DTaP) is recommended at ages 2, 4, and 6 months (doses 1–3); with booster doses at 15–18 months (dose 4) and 4–6 years (dose 5).^1^ The fifth dose of DTaP is not necessary if dose 4 was administered on or after the fourth birthday.^2^ The number of reported cases of pertussis in the US has increased in recent years, from 7,867 in 2000 to 20,762 in 2015, with substantially higher numbers during outbreak years (e.g., 48,277 in 2012).^3^ Potential reasons for this increase include: increased awareness, better reporting, improved diagnostic tests, increased circulation of *Bordetella pertussis*, evolution of *Bordetella pertussis* to escape protective immunity, waning immunity, and asymptomatic transmission from vaccinated individuals.^4,5^ Another reason for the increase in pertussis could be suboptimal immunization.^5^ While this can be due to missed opportunities for vaccination,^6–9^ various studies have shown that some parents choose to delay or refuse child vaccination.^10–15^ For example, Smith et al.^10^ report that 25.8% of 11,206 parents of children aged 24–35 months delayed, 8.2% refused, and 5.8% delayed and refused vaccines in 2009. Either way, undervaccination has been shown to leave children at an increased risk of pertussis infection.^16–18^ This is supported by US data that have linked clusters of non-medical exemptions (e.g., for religious reasons) with pertussis clusters.^19,20^

The yearly US National Immunization Survey (NIS) estimated that, during 2008–2012, 94–96% of children aged 19–35 months received ≥ 3 doses of DTaP, and 83–85% received ≥ 4 doses.^21^ However, it did not examine vaccination ages or uptake of the fifth DTaP dose at age 4–6 years. Non-compliance (i.e., receiving doses later than recommended) may at least partly explain the discrepancy between the reported high DTaP completion rates^21^ and the increased incidence of pertussis.^22^

To the best of our knowledge, there are no studies on compliance or completion up to the fifth DTaP dose. Therefore, the objectives of the current study were to: (1) measure DTaP compliance (correct timing of doses) and completion (within more lenient time frames) – to 3, 4, and 5 doses – among commercially insured and Medicaid-enrolled children; and (2) identify predictors of DTaP compliance and completion.

## Methods

### Data sources

This study (GSK study identifier: HO-14–14383) used data from the January 2005 to December 2013 *Truven Health MarketScan* Commercial and Multi-State Medicaid databases, which were analyzed separately. These databases are the gold standard in proprietary US research databases^23^ and have supported research in over 1000 peer-reviewed articles including research published by the Centers for Disease Control and Prevention (CDC).^24–26^ The Commercial database covers more than 25% of US employer-sponsored insurance beneficiaries; the Medicaid database covers 11 geographically diverse small and large states. All database records were anonymized and fully compliant with US patient confidentiality requirements, including the Health Insurance Portability and Accountability Act of 1996.

### Study population

The study included all children born during 2005–2011 with ≥ 2 years of continuous enrollment beginning at birth whose data could be linked to their birth record. Children whose date of birth did not match the start date of continuous enrollment were excluded, but there were no exclusions based on contraindications to DTaP. This population provided data on completion and compliance for the first 4 doses. A subsample of children with continuous enrollment from birth until their seventh birthday provided data for the fifth dose.

### Definitions and outcomes

The Advisory Committee on Immunization Practices (ACIP)-recommended ages for DTaP vaccination are shown in Table 1.^1^ Children were “dose compliant” if they received their dose ≤ 4 days before the ACIP minimum age (as per ACIP guidelines)^1^ and ≤ 1 month after the ACIP recommended age (or ≤ 1 year after for the fifth dose) (Table 1).^27^ “Series compliance” up to doses 3, 4, and 5 was defined as the child being dose compliant for each dose up to and including the relevant dose. “Series completion” to doses 1–3, 1–4, and 1–5 was defined using more lenient age limits (8 months, 24 months, and seventh birthday, respectively; Table 1). Children could be series complete to doses 1–4 and 1–5 regardless of the timing of doses 1–3 and 1–4, respectively. Other outcomes were the proportions of children receiving the third and fourth doses, regardless of timing; and the ages and time delays of those with delayed third and fourth doses. Of note, we used “compliant” to indicate vaccination according to guidelines, but other studies have used terms such as “on-time” or “age-appropriate”.

**Table 1.** ACIP recommended ages for DTaP vaccination and limits used to define compliance and completion.

DTaP dose	Recommended age (ACIP^1^)	Minimum age (ACIP^1^)	Age limits for compliance^a 1,27^	Age limits for completion^b^
1	2 months	6 weeks	38–92 days (approx. 1.2–3.0 months)	–
2	4 months	10 weeks	66–153 days (approx. 2.2–5.0 months)	–
3	6 months	14 weeks	94–214 days (approx. 3.1–7.0 months)	Doses 1–3 by 8 months
4	15–18 months	12 months	361–579 days (approx. 11.9–19.0 months)	Doses 1–4 by 24 months
5	4–6 years	4 years	1457–2556 days (approx. 4.0–7.0 years)	Doses 1–5 before seventh birthday

*ACIP* Advisory Committee on Immunization Practices, *DTaP* diphtheria, tetanus, and acellular pertussis vaccine

^a^In order to be considered series compliant, the child had to receive each dose within the recommended age limits (≤ 4 days before the minimum age to ≤ 1 month after the recommended age [≤ 1 year for the fifth dose])

^b^To be considered series complete to doses 1–4 or 1–5, the timing of doses 1–3 or 1–4, respectively, was not important, provided dose 4 or 5 was received by age 24 months or seventh birthday, respectively

Days delayed were calculated for each non-compliant child using a method outlined by Luman et al.^27^ Receipt of DTaP included all brands that provide immunization against diphtheria, tetanus, and acellular pertussis. Compliance and completion outcomes were measured based on administrative claims for the receipt of any DTaP injection, regardless of brand or brand switching. Claims for more than one DTaP dose on the same day were counted as one dose. Prescription drug claims that were independent from a claim for the administration of the vaccine (> 1 day between claims) were considered invalid and were excluded from DTaP dose counts.

### Statistical analysis

The MarketScan Commercial and Multi-State Medicaid databases were analyzed separately. Due to various differences between these two databases, we did not attempt to statistically compare the results. Categorical variables are shown as numbers (percentages), continuous data as means ± standard deviations (SDs). Binary indicators of series compliance and completion (for doses 1–3, 1–4, and 1–5) were assessed as a function of baseline demographic and clinical characteristics using multivariable logistic regressions with a binomial distribution and logit link. Odds ratios (ORs) and 95% confidence intervals (CIs) are reported. Data from children with ≥ 2 years continuous enrollment beginning at birth were used for models predicting compliance and completion for doses 1–4. The subsample of children with ≥ 7 years continuous enrollment was used for the fifth dose. Variables considered in the models included: year of birth, gender, neonatal intensive care unit (NICU), hospital stay, and birth hospitalization length of stay (LOS) (commercial and Medicaid); geographic region and household income based on zip code (commercial only); basis of Medicaid eligibility and race/ethnicity (Medicaid only).

Variables were considered significant independent predictors when the estimated 95% CI did not include 1.0 adjusted for all other variables included in the model. C-statistics were calculated for the models; 0.5–0.7 indicates poor predictive value, 0.7–0.8 reasonable predictive value, and ≥ 0.8 good predictive value.^28^

## Results

### Study population

Data for 1,914,364 commercially insured and 1,716,566 Medicaid-enrolled children born during 2005–2011 were extracted from the respective databases (Additional file 1: Fig. S1). Approximately 99% of these could be linked to the child’s birth record. Of these, there were 367,493 commercially insured and 766,153 Medicaid-enrolled children with ≥ 2 years of follow-up from birth; and 23,574 commercially insured and 41,284 Medicaid-enrolled children with ≥ 7 years of follow-up from birth. Baseline data for the commercial and Medicaid populations are shown in Table 2. The numbers of included children increased from 2005 to 2010, but was lower in 2011. Baseline characteristics of the subgroup of children with ≥ 7 years of follow-up were generally similar to those with ≥ 2 years of follow-up, although those with longer follow-up had a later mean age at first DTaP vaccination. However, this was due to outliers, as the median [interquartile range] ages were similar (commercial: 64 [61–70] and 64 [61–71] months; Medicaid: 68 [63–100] and 70 [63–128] months for ≥ 2 and ≥ 7 years of follow-up, respectively).

**Table 2.** Baseline characteristics.

	≥ 2 years follow-up		≥ 7 years follow-up	
	Commercial (*n* = 367,493)	Medicaid (*n* = 766,153)	Commercial (*n* = 23,574)	Medicaid (*n* = 41,284)
Birth year, *n* (%)^a^				
2005	26,231 (7.1)	70,734 (9.2)	10,333 (43.8)	26,896 (65.1)
2006	35,684 (9.7)	71,057 (9.3)	13,241 (56.2)	14,388 (34.9)
2007	47,506 (12.9)	87,140 (11.4)	–	–
2008	56,351 (15.3)	150,392 (19.6)	–	–
2009	66,319 (18.0)	156,550 (20.4)	–	–
2010	69,763 (19.0)	153,431 (20.0)	–	–
2011	65,639 (17.9)	76,849 (10.0)	–	–
Age at first DTaP vaccination, mean ± SD, days	104 ± 198	162 ± 298	160 ± 365	263 ± 484
Male, *n* (%)	189,884 (51.7)	392,100 (51.2)	12,266 (52.0)	21,373 (51.8)
Geographic region,^a,b^ *n* (%)				
South	153,524 (41.8)	–	11,464 (48.6)	–
Northeast	65,828 (17.9)	–	4059 (17.2)	–
West	42,066 (11.4)	–	1426 (6.0)	–
Midwest	98,963 (26.9)	–	6452 (27.4)	–
Unknown	7112 (1.9)	–	173 (0.7)	–
Household income,^c^ mean ± SD, $	45,106 ± 19,830	–	44,565 ± 18,865	–
Race/ethnicity,^a^ *n* (%)				
White	–	357,333 (46.6)	–	21,178 (51.3)
Black	–	263,941 (34.5)	–	16,470 (39.9)
Hispanic	–	74,809 (9.8)	–	1605 (3.9)
Other^d^	–	18,029 (2.4)	–	652 (1.6)
Unknown	–	52,041 (6.8)	–	1379 (3.3)
NICU hospital stay at birth, *n* (%)	35,381 (9.6)	73,095 (9.5)	2084 (8.8)	4625 (11.2)
Birth hospitalization length of stay				
Mean ± SD, days	3.9 ± 9.2	4.1 ± 10.3	3.8 ± 8.4	5.0 ± 12.8
≤ 2 days, *n* (%)	237,986 (64.8)	532,534 (69.5)	15,243 (64.7)	27,043 (65.5)
3–4 days, *n* (%)	93,035 (25.3)	157,771 (20.6)	6068 (25.7)	9027 (21.9)
5–6 days, *n* (%)	8653 (2.4)	16,737 (2.2)	573 (2.4)	1084 (2.6)
7–13 days, *n* (%)	12,974 (3.5)	25,177 (3.3)	803 (3.4)	1664 (4.0)
≥ 14 days, *n* (%)	14,845 (4.0)	33,934 (4.4)	887 (3.8)	2466 (6.0)

*DTaP* diphtheria, tetanus, and acellular pertussis vaccine, *NICU* neonatal intensive care unit, *SD* standard deviation

^a^Sum of percentages may not equal 100.0% due to rounding

^b^United States Census Bureau geographic region of residence for primary payer

^c^Median household income of the zipcode of the primary payer residence

^d^American Indian or Alaska Native, Native Hawaiian or Other Pacific Islands, or two or more races

### Completion and compliance

**Figure 1.**
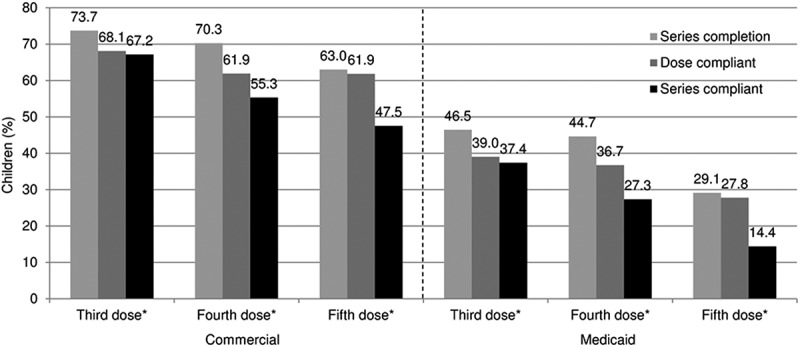
DTaP series completion, dose compliance, and series compliance rates among commercially insured and Medicaid-enrolled children. *DTaP* diphtheria, tetanus, and acellular pertussis vaccine *Third and fourth doses were measured among those in the 2-year follow-up cohorts (commercial, *n* = 367,493; Medicaid, *n* = 766,153); fifth dose among those in the 7-year follow-up cohorts (commercial, *n *= 23,574; Medicaid, *n* = 41,284)\

Series completion ranged from 73.7% (doses 1–3) to 63.0% (doses 1–5) among commercially insured children; and from 46.5% to 29.1% among Medicaid-enrolled children (Figure 1). Series compliance was lower: 67.2% (doses 1–3) to 47.5% (doses 1–5) among commercially insured children; and 37.4% to 14.4% among Medicaid-enrolled children (Figure 1).

**Figure 2.**
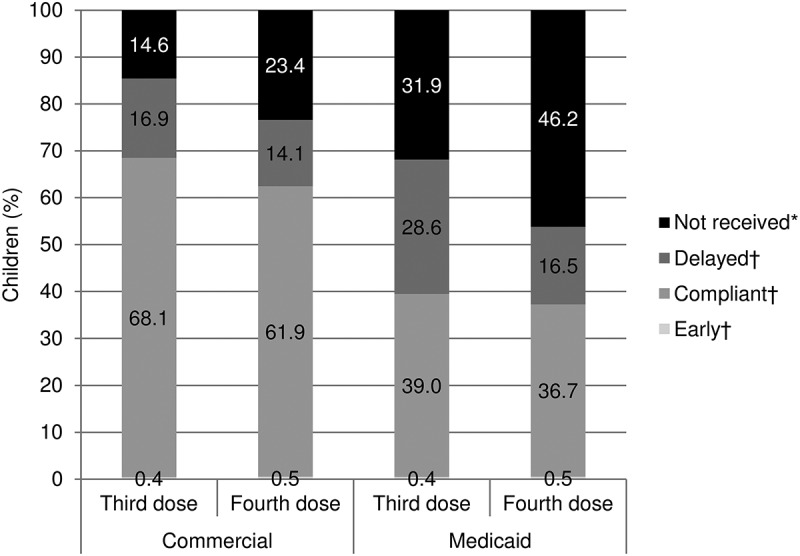
DTaP diphtheria, tetanus, and acellular pertussis vaccine(DTaP) third and fourth dose receipt status by the end of follow-up. *DTaP* diphtheria, tetanus, and acellular pertussis vaccine Sum of percentages may not equal 100.0% due to rounding *For children with ≥ 2 years of follow-up †Please see Table 1 for compliance timeframes

Overall, 68.1% of commercially insured children received the third DTaP dose on time, 16.9% received it late, 0.4% received it early, and 14.6% did not receive it during the study follow-up; results were somewhat worse for the fourth dose (Figure 2). Among Medicaid-enrolled children, 39.0% received the third dose on time, 28.6% late, 0.4% early, and 31.9% did not receive it during follow-up; again, results were worse for the fourth dose (Figure 2).

Among commercially insured children, 85.4% received a third dose of DTaP, at a mean age of 7.8 months; and 76.6% received a fourth dose, at a mean age of 18.5 months (Table 3). Among Medicaid-enrolled children, 68.1% received a third dose, at a mean age of 10.2 months; and 53.8% received a fourth dose, at a mean age of 20.7 months. Approximately 14–29% of children had delayed third/fourth vaccinations, with mean delays ranging from 7.1 to 12.0 months (Table 3).

**Table 3.** DTaP receipt and delays among children with ≥ 2 years follow-up.

	Commercial (*n* = 367,493)	Medicaid (*n* = 766,153)
Children who received a third dose,^a^ *n* (%)	313,901 (85.4)	521,938 (68.1)
Age at third dose receipt, mean ± SD, months	7.8 ± 5.5	10.2 ± 8.5
Children who received a fourth dose,^a^ *n* (%)	281,435 (76.6)	412,194 (53.8)
Age at fourth dose receipt, mean ± SD, months	18.5 ± 7.3	20.7 ± 10.2
Children with delayed third vaccination (age > 214 days), *n* (%)	62,048 (16.9)	219,411 (28.6)
Delayed vaccination,^b^ mean ± SD, months	7.1 ± 10.1	8.6 ± 11.1
Children with delayed fourth vaccination (age > 579 days), *n* (%)	51,890 (14.1)	126,607 (16.5)
Delayed vaccination,^b^ mean ± SD, months	9.2 ± 12.6	12.0 ± 13.3

*DTaP* diphtheria, tetanus, and acellular pertussis vaccine, *SD* standard deviation

^a^Measured among children who received the dose at any time during the follow-up period, regardless of timing

^b^Measured among children with delayed vaccination

### Predictors of completion and compliance

**Figure 3.**
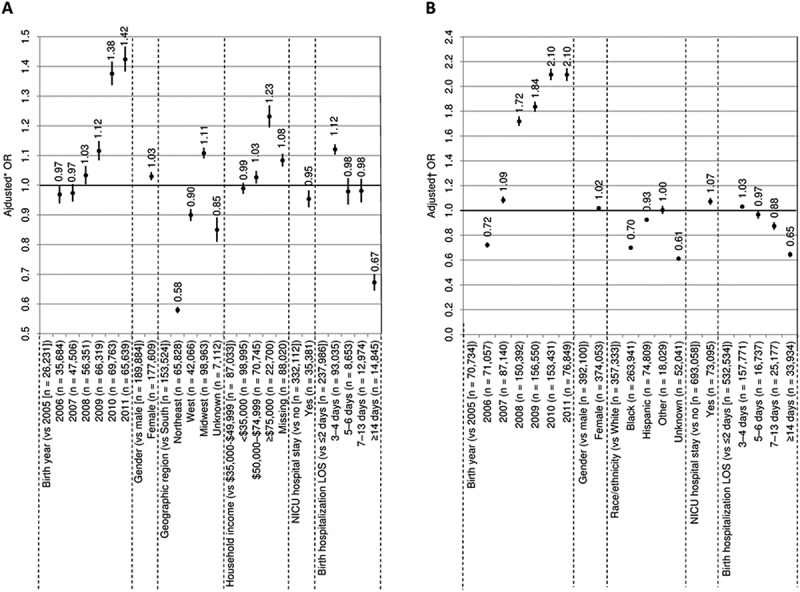
Predictors of DTaP series compliance for doses 1–4 among commercially insured (A) and Medicaid-enrolled (B) children.CI confidence interval, DTaP diphtheria, tetanus, and acellular pertussis vaccine, LOS length of stay, NICU neonatal intensive care unit, OR odds ratio Bars show 95% CIs *ORs were adjusted for birth year, gender, geographic region, household income, NICU hospital stay, and birth hospitalization LOS †ORs were adjusted for birth year, gender, race/ethnicity, NICU hospital stay, birth hospitalization LOS, and basis of Medicaid eligibility

Among commercially insured children, the strongest predictors of series compliance to doses 1–4 were later birth year (2011 or 2010) and household income > $75,000; the strongest predictors of non-compliance were Northeast geographic region and birth hospitalization LOS ≥ 14 days (Figure 3A; Additional file 2: Table S1). Predictors of series compliance to doses 1–3 and 1–5 were similar to those for 1–4 (Additional file 2: Table S1). Predictors of series completion to 3, 4, and 5 doses were also generally similar, although household income > $75,000 had a larger beneficial influence on 5-dose completion (Additional file 2: Table S1).

Among Medicaid-enrolled children, later birth year (2011 or 2010) was the strongest predictor of series compliance to doses 1–4; the strongest predictors of non-compliance were hospitalization LOS ≥ 14 days and Black race/ethnicity (Figure 3B; Additional file 3: Table S2). Predictors of series compliance to doses 1–3 and 1–5 were mainly similar; as were predictors of series completion to 3, 4, and 5 doses (Additional file 3: Table S2). The only notable differences were that Black and Hispanic race/ethnicities had a greater impact on 5-dose compliance and completion.

C-statistics were 0.575–0.623 for the six commercial models and 0.606–0.623 for the six Medicaid models (Additional files 2 and 3: Tables S1 and S2), hence these regression results should be interpreted with caution.

## Discussion

Among 367,493 commercially insured children, series compliance and completion rates for DTaP doses 1–4 were 55.3% and 70.3%, respectively; among 766,153 Medicaid-enrolled children, the respective rates were 27.3% and 44.7%. Although we did not compare the commercial and Medicaid results statistically, nor adjust for confounding factors (most importantly that some Medicaid-enrolled children likely received vaccines through the Vaccines for Children Program, which would not have been captured), differences in vaccine compliance/completion between the two populations have been found in other studies. For example, Smith et al.^29^ analyzed data from 8324 children aged 19–24 months from NIS 2001/2002, and estimated that 75.6% of privately insured and 70.0% of children covered by Medicaid/State Children’s Health Insurance Program were up to date with their recommended vaccinations. Similarly, among 735 children aged 19–35 months, publicly-insured children were less likely to be up to date with vaccinations than privately-insured children before adjustment for confounders (OR, 0.3; 95% CI, 0.2–0.6), but not after adjustment (OR 0.6; 95% CI, 0.3–1.2).^30^

In the current study, 70.3% of commercially insured children and 44.7% of Medicaid-enrolled children received doses 1–4 by age 24 months. However, in the 2012 NIS, 82.5% (95% CI, 81.3–83.7) of children aged 19–35 months were estimated to have completed ≥ 4 doses.^21^ The discrepancy between our rates and that reported by the NIS is likely due to the very different methodologies used, the different data sources, and the different ages when coverage was assessed.

Despite these discrepancies and the potential underestimation of completion in the current study, our results clearly show that DTaP vaccination was delayed for many of those who received it. Among commercially insured children, 18.4% of those who received a fourth dose received it late (age > 19 months); among Medicaid-enrolled children, this was 30.7%. The mean delays were 9 and 12 months for the two groups, respectively. Given that infants aged < 1 year have the highest reported pertussis incidence,^31^ such delays could leave infants at increased risk of infection.^16^

In the current study, the strongest predictor of DTaP compliance/completion was later birth year, indicating that they improved over time. This may be due to interventions to improve pediatric vaccination coverage (e.g., provider persistence^32^ and combination vaccines^33-35^) or increased awareness due to outbreaks. However, this trend was not apparent in NIS data during 2005–2012,^21,36^ possibly because the NIS rates were consistently very high.

Higher household income (particularly ≥ $75,000) predicted improved compliance/completion (among commercially insured children). This is in line with a study from 1995–2007 that reported significantly higher DTaP/diphtheria and tetanus toxoids and whole-cell pertussis (DTwP) timely vaccination among children of families earning ≥ 134% versus ≤ 133% of the federal poverty level.^37^ Similarly, the NIS 2012 reported that children living at or above versus below the poverty line were more likely to receive ≥ 4 doses (85.0% vs. 78.5%).^21^

Black and Hispanic race/ethnicities predicted poorer compliance/completion (among Medicaid-enrolled children). This trend was also found in the NIS 2012 data: Black and Hispanic children were less likely than White children to receive ≥ 4 doses (79.6% and 80.8% vs. 83.6%).^21^ Similarly, Santoli et al.^30^ reported that Black and Hispanic children were less likely to be up to date with vaccinations than White children. Luman et al.^27^ reported that Black children were more likely to have severely delayed vaccination than White children, but that Hispanic children were less likely to have severely delayed vaccination than White children. Dempsey et al.,^11^ however, reported that Black parents were more likely to follow the recommended vaccination schedule than White parents; while Smith et al.^15^ reported that undervaccination was associated with Black children, but unvaccinated children were more likely to be white.

## Limitations

The MarketScan Research Databases are based on administrative claims and – as with all data of this nature – are subject to data coding limitations and data entry error. Furthermore, it should be noted that some Medicaid-enrolled children may have received DTaP vaccination via the Vaccines for Children Program, which would not result in a claim and would therefore not have been captured in this study. There is also potential for misclassification of covariates or study outcomes as children were identified through administrative claims data rather than medical records. Given the discrepancy between our real-world claims database methodology and that from the NIS,^21^ it is not surprising that completion results appear to be an underestimation in the current analysis. However, our study clearly shows that, among those who received DTaP vaccination, many doses were delayed.

As this study was limited to children included in the MarketScan databases with commercial or Medicaid health coverage and ≥ 2 (or ≥ 7) years of follow-up, the findings may not be representative of the entire US population. Also, children with long-term Medicaid coverage are not necessarily representative of those covered for shorter periods. Further, Medicaid data were only available for 11 small and large states and were not stratified by state, which may affect the external validity of results as some states receive and allocate federal vaccination funding differently than others. Furthermore, the Universal Vaccine Purchasing Program, through which certain states purchase vaccines for all children regardless of Medicaid eligibility, may be associated with higher vaccination coverage in the participating states compared to states without such purchasing policies.^38^

ACIP recommends the fourth DTaP dose by 18 months, but package inserts for *Infanrix* (GSK, Belgium)^39^ and *Daptacel* (Sanofi Pasteur, USA)^40^ recommend 15–20 months of age, hence children who were vaccinated at 19–20 months would have been compliant according to the package insert, but not in our study (although they would have met our “completion” criteria). Delays to the fifth dose (after the child’s seventh birthday) are not reported as we only examined DTaP administration, and the catch-up vaccination recommendation for children aged ≥ 7 years is tetanus toxoid, reduced diphtheria toxoid, and acellular pertussis (Tdap) or adult tetanus and diphtheria toxoids (Td).^1^ It should also be noted that, while most states require 4–5 doses of DTaP, others require fewer doses.^41^ This may have impacted on coverage, particularly to the fifth dose, and compliance/completion by geographic region. Further, the minimum intervals between DTaP doses were not included in the calculations of compliance, nor did we take into account that children who receive their fourth dose on or after their fourth birthday do not require a fifth dose at age 4–6 years.^2^ Lastly, it should be noted that, while we report results for commercially insured and Medicaid-enrolled children, we did not attempt to statistically compare the results between these two populations as there are many potentially confounding factors that were not taken into account.

## Conclusions

While improvements in DTaP series compliance were observed over time in this large, comprehensive study, overall, we found that compliance to the first 4 doses was low, with many children receiving delayed doses, which were often many months late. Compliance to the fifth dose was even poorer than for the first 4 doses. As such delays could leave many infants and children at risk of pertussis infection, education programs to increase awareness of the importance of timely vaccination may be beneficial.

## Data Availability

This study used data from the 2005–2013 *Truven Health MarketScan* Commercial Database and the Multi-State Medicaid Database. These databases are the property of Truven Health Analytics and their use for this study was licensed by GSK; they are not able to be made publicly available.

## Declarations

**Ethics approval and consent to participate**

This study was exempt from the review by an Institutional Review Board per United States Department of Health and Human Services (HHS) regulation for the protection of human subjects in research (Title 45 of the Code of Federal Regulations [CFR], Part 46.101(b)(4), made available by the Office for Human Research Protections [OHRP] at *www.hhs.gov/ohrp/regulations-and-policy/regulations/45-cfr-46*), for two main reasons: (1) the study is a retrospective analysis of existing data (hence no patient intervention or interaction), (2) no patient-identifiable information are included in the dataset.

## Trademark statement

*Infanrix* is a trademark of the GSK group of companies.

*Daptacel* is a trademark of Sanofi Pasteur.

## Abbreviations

ACIP: Advisory Committee on Immunization Practices
CI: confidence interval
DTaP: diphtheria, tetanus, and acellular pertussis vaccine
DTwP: diphtheria, tetanus, and whole-cell pertussis vaccine
HMO: health maintenance organization;
LOS: length of stay
NCQA: National Committee for Quality Assurance
NICU: neonatal intensive care unit;
NIS: National Immunization Survey
OR: odds ratio
PPO: preferred provider organization
SD: standard deviation
Td: adult tetanus and diphtheria vaccine
Tdap: reduced antigen content tetanus, diphtheria, and acellular pertussis vaccine
US: United States

## References

[1] Kroger AT, Sumaya CV, Pickering LK, Atkinson WL. General recommendations on immunization: recommendations of the Advisory Committee on Immunization Practices (ACIP). MMWR Recomm Rep. 2011;60(2):1–64. 21293327

[2] Centers for Disease Control and Prevention (CDC) Pertussis: summary of vaccine recommendations. [accessed 2017 May 3]. https://www.cdc.gov/vaccines/vpd/pertussis/recs-summary.html#note1.

[3] Centers for Disease Control and Prevention (CDC) Pertussis cases by year (1922-2015). [accessed 2017 5 3]. https://www.cdc.gov/pertussis/surv-reporting/cases-by-year.html#modalIdString_CDCTable_0.

[4] Centers for Disease Control and Prevention (CDC) Pertussis frequently asked questions. [accessed 2015 Dec 14]. http://www.cdc.gov/pertussis/about/faqs.html.

[5] Althouse BM, Scarpino SV Asymptomatic transmission and the resurgence of Bordetella pertussis. BMC Med. 2015;13:146. doi:10.1186/s12916-015-0382-8. 26103968 PMC4482312

[6] Robison SG Incomplete Early Childhood Immunization Series And Missing Fourth DTaP immunizations; missed opportunities or missed visits? ISRN Prev Med. 2013;2013:351540. doi:10.5402/2013/351540. 24967133 PMC4062864

[7] Irigoyen MM, Findley S, Wang D, Chen S, Chimkin F, Pena O, Mendonca E Challenges and successes of immunization registry reminders at inner-city practices. Ambul Pediatr. 2006;6(2):100–104. doi:10.1016/j.ambp.2005.10.006. 16530147

[8] Dodd D Benefits of combination vaccines: effective vaccination on a simplified schedule. Am J Manag Care. 2003;9(1 Suppl):S6–12. 12564784

[9] Tickner S, Leman PJ, Woodcock A Factors underlying suboptimal childhood immunisation. Vaccine. 2006;24(49–50):7030–7036. doi:10.1016/j.vaccine.2006.06.060. 16890330

[10] Smith PJ, Humiston SG, Marcuse EK, Zhao Z, Dorell CG, Howes C, Hibbs B Parental delay or refusal of vaccine doses, childhood vaccination coverage at 24 months of age, and the health belief model. Public Health Rep. 2011;126(Suppl 2):135–146. doi:10.1177/00333549111260S215. 21812176 PMC3113438

[11] Dempsey AF, Schaffer S, Singer D, Butchart A, Davis M, Freed GL Alternative vaccination schedule preferences among parents of young children. Pediatrics. 2011;128(5):848–856. doi:10.1542/peds.2011-0400. 21969290

[12] Glanz JM, Newcomer SR, Narwaney KJ, Hambidge SJ, Daley MF, Wagner NM, McClure DL, Xu S, Rowhani-Rahbar A, Lee GM A population-based cohort study of undervaccination in 8 managed care organizations across the United States. JAMA Pediatr. 2013;167(3):274–281. doi:10.1001/jamapediatrics.2013.502. 23338829

[13] Larson HJ, Jarrett C, Eckersberger E, Smith DM, Paterson P Understanding vaccine hesitancy around vaccines and vaccination from a global perspective: a systematic review of published literature, 2007-2012. Vaccine. 2014;32(19):2150–2159. doi:10.1016/j.vaccine.2014.01.081. 24598724

[14] Leib S, Liberatos P, Edwards K Pediatricians’ experience with and response to parental vaccine safety concerns and vaccine refusals: a survey of Connecticut pediatricians. Public Health Rep. 2011;126(Suppl 2):13–23. doi:10.1177/00333549111260S203. PMC3113426 21812165

[15] Smith PJ, Chu SY, Barker LE Children who have received no vaccines: who are they and where do they live? Pediatrics. 2004;114(1):187–195. 15231927 10.1542/peds.114.1.187

[16] Glanz JM, Narwaney KJ, Newcomer SR, Daley MF, Hambidge SJ, Rowhani-Rahbar A, Lee GM, Nelson JC, Naleway AL, Nordin JD Association between undervaccination with diphtheria, tetanus toxoids, and acellular pertussis (DTaP) vaccine and risk of pertussis infection in children 3 to 36 months of age. JAMA Pediatr. 2013;167(11):1060–1064. doi:10.1001/jamapediatrics.2013.2353. 24019039

[17] Glanz JM, McClure DL, Magid DJ, Daley MF, France EK, Salmon DA, Hambidge SJ Parental refusal of pertussis vaccination is associated with an increased risk of pertussis infection in children. Pediatrics. 2009;123(6):1446–1451. doi:10.1542/peds.2008-2150. 19482753

[18] Goldstein ND, Newbern EC, Evans AA, Drezner K, Welles SL Choice of measures of vaccination and estimates of risk of pediatric pertussis. Vaccine. 2015;33(32):3970–3975. doi:10.1016/j.vaccine.2015.06.033. 26093200

[19] Atwell JE, Van Otterloo J, Zipprich J, Winter K, Harriman K, Salmon DA, Halsey NA, Omer SB Nonmedical vaccine exemptions and pertussis in California, 2010. Pediatrics. 2013;132(4):624–630. doi:10.1542/peds.2013-0878. 24082000

[20] Omer SB, Enger KS, Moulton LH, Halsey NA, Stokley S, Salmon DA Geographic clustering of nonmedical exemptions to school immunization requirements and associations with geographic clustering of pertussis. Am J Epidemiol. 2008;168(12):1389–1396. doi:10.1093/aje/kwn263. 18922998

[21] Centers for Disease Control and Prevention (CDC) National, state, and local area vaccination coverage among children aged 19-35 months - United States, 2012. MMWR Morb Mortal Wkly Rep. 2013;62(36):733–740. 24025754 PMC4585572

[22] Adams DA, Jajosky RA, Ajani U, Kriseman J, Sharp P, Onwen DH, Schley AW, Anderson WJ, Grigoryan A, Aranas AE, et al Summary of notifiable diseases - United States, 2012. MMWR Morb Mortal Wkly Rep. 2014;61(53):1–121. 25233134

[23] Truven Health Analytics Truven health MarketScan Research Databases. [accessed 2017 5 3]. https://truvenhealth.com/Portals/0/assets/HP_11517_0912_MarketScanResearchDatabasesForHP_SS_WEB.pdf.

[24] Flannery B, Reynolds SB, Blanton L, Santibanez GA, O’Halloran A, Lu P-J, Chen J, Foppa IM, Gargiullo P, Bresee J, et al Influenza vaccine effectiveness against pediatric deaths: 2010–2014. Pediatrics. 2017;139(5):e20164244. doi:10.1542/peds.2016-4244. 28557757 PMC5728382

[25] Cortes JE, Curns AT, Tate JE, Cortese MM, Patel MM, Zhou F, Parashar UD Rotavirus vaccine and health care utilization for diarrhea in U.S. children. N Engl J Med.. 2011;365(12):1108–1117. doi:10.1056/NEJMoa1000446. 21992123

[26] Grosse SD, Boulet SL, Grant AM, Hulihan MM, Faughnan ME The use of US health insurance data for surveillance of rare disorders: hereditary hemorrhagic telangiectasia. Genet Med. 2014;16(1):33–39. doi:10.1038/gim.2013.66. 23703685 PMC4453870

[27] Luman ET, Barker LE, Shaw KM, McCauley MM, Buehler JW, Pickering LK Timeliness of childhood vaccinations in the United States: days undervaccinated and number of vaccines delayed. JAMA. 2005;293(10):1204–1211. doi:10.1001/jama.293.10.1204. 15755943

[28] Hosmer DW, Lemeshow SA Applied logistic regression. 2nd ed. New York (NY): John Wiley & Sons; 2000.

[29] Smith PJ, Stevenson J, Chu SY Associations between childhood vaccination coverage, insurance type, and breaks in health insurance coverage. Pediatrics. 2006;117(6):1972–1978. doi:10.1542/peds.2005-2414. 16740838

[30] Santoli JM, Huet NJ, Smith PJ, Barker LE, Rodewald LE, Inkelas M, Olson LM, Halfon N Insurance status and vaccination coverage among US preschool children. Pediatrics. 2004;113(6 Suppl):1959–1964. 15173467

[31] Centers for Disease Control and Prevention (CDC) Reported pertussis incidence by age group: 1990-2016. [accessed 2018 5 17]. https://www.cdc.gov/pertussis/images/incidence-graph-age.png.

[32] Opel DJ, Heritage J, Taylor JA, Mangione-Smith R, Salas HS, Devere V, Zhou C, Robinson JD The architecture of provider-parent vaccine discussions at health supervision visits. Pediatrics. 2013;132(6):1037–1046. doi:10.1542/peds.2013-2037. 24190677 PMC3838535

[33] Happe LE, Lunacsek OE, Kruzikas DT, Marshall GS Impact of a pentavalent combination vaccine on immunization timeliness in a state Medicaid population. Pediatr Infect Dis J. 2009;28(2):98–101. doi:10.1097/INF.0b013e318187d047. 19148039

[34] Happe LE, Lunacsek OE, Marshall GS, Lewis T, Spencer S Combination vaccine use and vaccination quality in a managed care population. Am J Manag Care. 2007;13(9):506–512. 17803364

[35] Marshall GS, Happe LE, Lunacsek OE, Szymanski MD, Woods CR, Zahn M, Russell A Use of combination vaccines is associated with improved coverage rates. Pediatr Infect Dis J. 2007;26(6):496–500. doi:10.1097/INF.0b013e31805d7f17. 17529866

[36] Centers for Disease Control and Prevention (CDC) National, state, and local area vaccination coverage among children aged 19-35 months - United States, 2007. MMWR Morb Mortal Wkly Rep. 2008;57(35):961–966. 18772851

[37] Smith PJ, Jain N, Stevenson J, Mannikko N, Molinari NA Progress in timely vaccination coverage among children living in low-income households. Arch Pediatr Adolesc Med. 2009;163(5):462–468. doi:10.1001/archpediatrics.2009.25. 19414693

[38] National Conference of State Legislatures (NCSL) Immunizations policy issues overview. [accessed 2015 April 28]. http://www.ncsl.org/research/health/immunizations-policy-issues-overview.aspx.

[39] GlaxoSmithKline Biologicals INFANRIX (Diphtheria and Tetanus Toxoids and Acellular Pertussis Vaccine Adsorbed). [accessed 2015 4 28] http://www.fda.gov/downloads/BiologicsBloodVaccines/Vaccines/ApprovedProducts/UCM124514.pdf.

[40] sanofi pasteur DAPTACEL (Diphtheria and Tetanus Toxoids and Acellular Pertussis Vaccine Adsorbed). [accessed 2015 April 28]. http://www.fda.gov/downloads/BiologicsBloodVaccines/Vaccines/ApprovedProducts/UCM103037.pdf.

[41] Centers for Disease Control and Prevention (CDC) Vaccination requirements for all grantees, for DTaP-Diphtheria, Tetanus, acellular Pertussis and Kindergarten. [accessed 2015 11 17]. http://www2a.cdc.gov/nip/schoolsurv/schImmRqmtReport.asp?s=grantee&d=4&w=WHERE%20a.gradeID=2%20AND%20a.vaccineID=1.

