# Supplementary material for: Age-appropriate compliance and completion of up to five doses of pertussis vaccine in US children
Source: Hum Vaccin Immunother. 2018 Aug 29;14(12):2932–9. doi: 10.1080/21645515.2018.1502526 (PMC6351022; doi:10.1080/21645515.2018.1502526)
Supplement: Supplemental Material [file khvi-14-12-1502526-s001.zip › KHVI_A_1502526_Supplemental 1.docx]

**Additional file 1: Fig. S1** Study flow chart


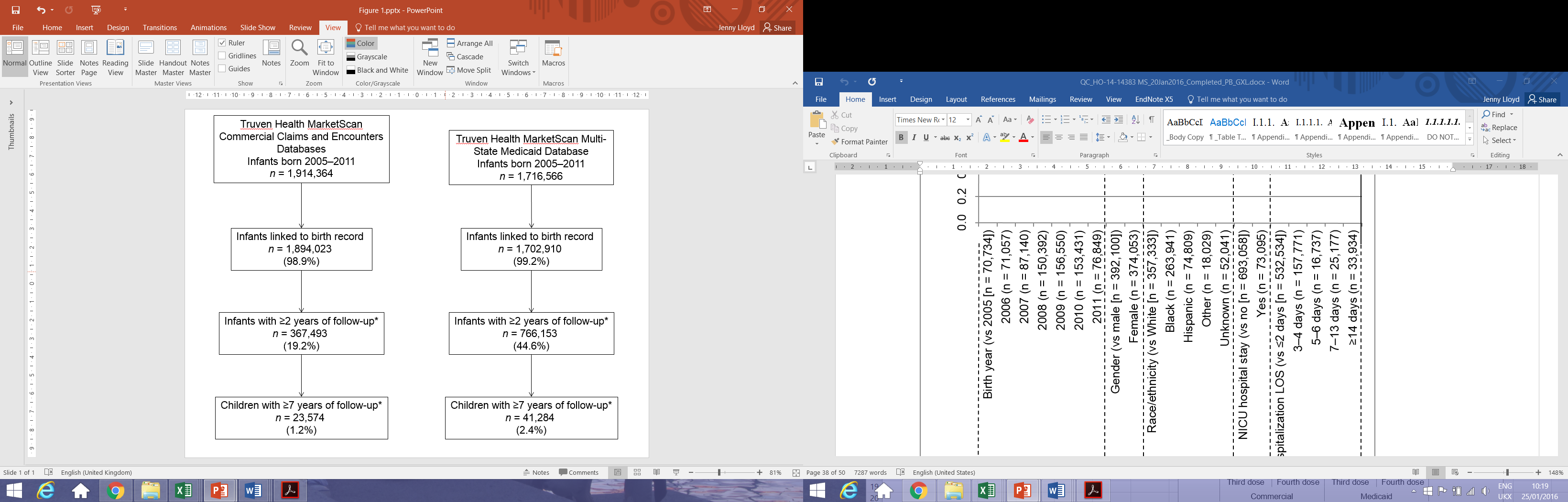


*Follow-up required continuous medical and pharmacy benefits enrollment beginning on the date of birth
