# Supplementary material for: Age-appropriate compliance and completion of up to five doses of pertussis vaccine in US children
Source: Hum Vaccin Immunother. 2018 Aug 29;14(12):2932–9. doi: 10.1080/21645515.2018.1502526 (PMC6351022; doi:10.1080/21645515.2018.1502526)
Supplement: Supplemental Material [file khvi-14-12-1502526-s001.zip › KHVI_A_1502526_Supplemental 2.docx]

**Additional file 2: Table S1** Commercial predictors of series compliance and completion

|  | Series Compliance^a^ | | | Completion | | |
| --- | --- | --- | --- | --- | --- | --- |
|  | 3 Doses | 4 Doses | 5 Doses | 3 Doses | 4 Doses | 5 Doses |
| C-statistic | 0.575 | 0.575 | 0.600 | 0.583 | 0.582 | 0.623 |
| Birth year (vs. 2005) |  |  |  |  |  |  |
| 2006 | 1.01  (0.97–1.04) | 0.97  (0.94–1.00) | 1.00  (0.95–1.05) | 1.02  (0.99–1.06) | 0.99  (0.96–1.03) | 1.09  (**1.03–1.15**) |
| 2007 | 1.00  (0.97–1.03) | 0.97  (0.95–1.00) | – | 1.05  (**1.01–1.08**) | 1.05  (**1.01–1.08**) | – |
| 2008 | 1.07  (**1.03–1.10**) | 1.03  (**1.00–1.07**) | – | 1.16  (**1.12–1.20**) | 1.18  (**1.14–1.21**) | – |
| 2009 | 1.12  (**1.08–1.15**) | 1.12  (**1.08–1.15**) | – | 1.19  (**1.15–1.23**) | 1.28  (**1.24–1.32**) | – |
| 2010 | 1.38  (**1.34–1.43**) | 1.38  (**1.34–1.42**) | – | 1.51  (**1.46–1.56**) | 1.58  (**1.53–1.63**) | – |
| 2011 | 1.51  (**1.46–1.56**) | 1.42  (**1.38–1.47**) | – | 1.69  (**1.64–1.75**) | 1.75  (**1.69–1.80**) | – |
| Sex (vs. male) |  |  |  |  |  |  |
| Female | 1.02  (**1.01–1.03**) | 1.03  (**1.02–1.04**) | 1.04  (0.99–1.10) | 1.02  (**1.00–1.03**) | 1.02  (**1.00–1.03**) | 1.00  (0.95–1.06) |
| Geographic region^b^ (vs. South) |  |  |  |  |  |  |
| Northeast | 0.59  (**0.58–0.60**) | 0.58  (**0.57–0.59**) | 0.39  (**0.36–0.42**) | 0.56  (**0.55–0.58**) | 0.60  (**0.59–0.61**) | 0.33  (**0.31–0.36**) |
| Midwest | 1.07  (**1.05–1.09**) | 1.11  (**1.09–1.13**) | 1.12  (**1.06–1.20**) | 1.05  (**1.03–1.07**) | 1.08  (**1.06–1.10**) | 1.14  (**1.06–1.22**) |
| West | 0.91  (**0.89–0.93**) | 0.90  (**0.88–0.92**) | 0.89  (**0.80–1.00**) | 0.88  (**0.86–0.90**) | 0.85  (**0.83–0.87**) | 0.89  (0.79–1.00) |
| Unknown | 0.80  (**0.77–0.85**) | 0.85  (**0.81–0.89**) | 0.86  (0.64–1.17) | 0.76  (**0.72–0.80**) | 0.76  (0.72–0.80) | 0.82  (0.60–1.12) |
| Household income^c^ (vs. $35 000–$49 999) |  |  |  |  |  |  |
| <$35 000 | 0.95  (**0.94–0.97**) | 0.99  (0.97–1.01) | 0.85  (**0.78–0.93**) | 0.93  (0.91–0.95) | 0.94  (0.92–0.96) | 0.79  (0.73–0.86) |
| $50 000–$74 999 | 1.03  (**1.01–1.05**) | 1.03  (**1.01–1.05**) | 1.18  (**1.07–1.30**) | 1.02  (1.00–1.05) | 1.06  (**1.03–1.08**) | 1.27  (**1.14–1.41**) |
| ≥$75 000 | 1.35  (**1.31–1.40**) | 1.23  (**1.20–1.27**) | 1.98  (**1.74–2.27**) | 1.46  (**1.41–1.52**) | 1.48  (**1.43–1.53**) | 3.13  (**2.68–3.66**) |
| Missing | 1.07  (**1.05–1.09**) | 1.08  (**1.06–1.11**) | 1.16  (**1.07–1.24**) | 1.08  (**1.06–1.11**) | 1.10  (**1.08–1.13**) | 1.13  (**1.05–1.22**) |
| NICU hospital stay (vs. no) |  |  |  |  |  |  |
| Yes | 0.96  (**0.93–0.99**) | 0.95  (**0.93–0.98**) | 1.07  (0.95–1.21) | 0.99  (0.95–1.02) | 0.98  (0.95–1.02) | 1.26  (**1.10–1.43**) |
| Birth hospitalization LOS (vs. ≤2 days) |  |  |  |  |  |  |
| 3–4 days | 1.11  (**1.09–1.13**) | 1.12  (**1.10–1.14**) | 1.06  (0.99–1.12) | 1.12  (**1.10–1.14**) | 1.14  (**1.12–1.16**) | 1.03  (0.97–1.10) |
| 5–6 days | 0.93  (**0.89–0.98**) | 0.98  (0.94–1.02) | 0.93  (0.78–1.12) | 0.95  (0.90–1.00) | 1.00  (0.96–1.05) | 0.88  (0.73–1.06) |
| 7–13 days | 0.97  (0.93–1.01) | 0.98  (0.94–1.02) | 0.94  (0.80–1.11) | 1.00  (0.95–1.05) | 1.04  (0.99–1.08) | 0.87  (0.73–1.03) |
| ≥14 days | 0.59  (**0.57–0.62**) | 0.67  (**0.65–0.70**) | 0.69  (**0.58–0.81**) | 0.57  (**0.54–0.59**) | 0.65  (**0.62–0.67**) | 0.65  (**0.55–0.77**) |

Data are adjusted^d^ ORs (95% CIs). **Bold** indicates 95% CIs not crossing 1.0 (significant at p <0.05 level). Child numbers varied according to whether the model analyzed 3, 4, or 5 doses. For child numbers in each group, please refer to Table 2

*CI* confidence interval, *LOS* length of stay, *NICU* neonatal intensive care unit, *OR* odds ratio

^a^Series compliance: age compliant for dose and all previous doses

^b^United States Census Bureau geographic region of residence for primary payer

^c^Median household income of the zipcode of the primary payer residence

^d^ORs were adjusted for birth year, gender, geographic region, household income, NICU hospital stay, and birth hospitalization LOS
