# Supplementary material for: Age-appropriate compliance and completion of up to five doses of pertussis vaccine in US children
Source: Hum Vaccin Immunother. 2018 Aug 29;14(12):2932–9. doi: 10.1080/21645515.2018.1502526 (PMC6351022; doi:10.1080/21645515.2018.1502526)
Supplement: Supplemental Material [file khvi-14-12-1502526-s001.zip › KHVI_A_1502526_Supplemental 3.docx]

**Additional file 3: Table S2** Medicaid predictors of series compliance and completion

|  | Series Compliance^a^ | | | Completion | | |
| --- | --- | --- | --- | --- | --- | --- |
|  | 3 Doses | 4 Doses | 5 Doses | 3 Doses | 4 Doses | 5 Doses |
| C-statistic | 0.610 | 0.606 | 0.623 | 0.622 | 0.621 | 0.611 |
| Birth year (vs. 2005) |  |  |  |  |  |  |
| 2006 | 0.64  (**0.63–0.66**) | 0.72  (**0.70–0.74**) | 0.68  (**0.64–0.72**) | 0.63  (**0.61–0.64**) | 0.72  (**0.71–0.74**) | 0.68  (**0.65–0.71**) |
| 2007 | 0.90  (**0.88–0.92**) | 1.09  (**1.06–1.11**) | – | 0.92  (**0.90–0.94**) | 1.19  (**1.17–1.22**) | – |
| 2008 | 1.48  (**1.46–1.51**) | 1.72  (**1.68–1.76**) | – | 1.55  (**1.52–1.58**) | 1.91  (**1.87–1.94**) | – |
| 2009 | 1.57  (**1.54–1.60**) | 1.84  (**1.80–1.88**) | – | 1.64  (**1.61–1.67**) | 2.03  (**1.99–2.07**) | – |
| 2010 | 1.84  (**1.81–1.88**) | 2.10  (**2.05–2.14**) | – | 1.99  (**1.95–2.02**) | 2.40  (**2.35–2.44**) | – |
| 2011 | 1.90  (**1.86–1.94**) | 2.10  (**2.05–2.15**) | – | 2.03  (**1.99–2.07**) | 2.36  (**2.31–2.41**) | – |
| Sex (vs. male) |  |  |  |  |  |  |
| Female | 1.02  (**1.01–1.03**) | 1.02  (**1.01–1.03**) | 1.02  (0.96–1.07) | 1.01  (**1.00–1.02**) | 1.00  (0.99–1.01) | 1.01  (0.97–1.05) |
| Race/ethnicity (vs. White) |  |  |  |  |  |  |
| Black | 0.70  (**0.69–0.71**) | 0.70  (**0.69–0.71**) | 0.48  (**0.46–0.52**) | 0.71  (**0.70–0.72**) | 0.78  (**0.78–0.79**) | 0.59  (**0.56–0.62**) |
| Hispanic | 0.89  (**0.87–0.90**) | 0.93  (**0.91–0.94**) | 0.27  (**0.22–0.33**) | 0.87  (**0.86–0.89**) | 0.86  (**0.85–0.87**) | 0.18  (**0.15–0.21**) |
| Other^b^ | 1.01  (0.98–1.05) | 1.00  (0.97–1.04) | 1.11  (0.91–1.35) | 1.04  (**1.01–1.08**) | 1.07  (**1.04–1.10**) | 1.15  (0.98–1.36) |
| Unknown | 0.58  (**0.57–0.59**) | 0.61  (**0.60–0.63**) | 0.59  (**0.49–0.70**) | 0.55  (**0.54–0.57**) | 0.57  (**0.56–0.59**) | 0.66  (**0.58–0.76**) |
| NICU hospital stay (vs. no) |  |  |  |  |  |  |
| Yes | 1.07  (**1.05–1.10**) | 1.07  (**1.05–1.10**) | 1.18  (**1.04–1.35**) | 1.08  (**1.06–1.11**) | 1.10  (**1.08–1.13**) | 1.24  (**1.12–1.37**) |
| Birth hospitalization LOS (vs. ≤2 days) |  |  |  |  |  |  |
| 3–4 days | 1.02  (**1.01–1.03**) | 1.03  (**1.02–1.05**) | 0.97  (0.91–1.04) | 1.01  (1.00–1.02) | 0.99  (0.98–1.01) | 0.96  (0.90–1.01) |
| 5–6 days | 0.95  (**0.92–0.98**) | 0.97  (0.93–1.01) | 0.93  (0.78–1.12) | 0.94  (**0.91–0.97**) | 0.94  (**0.90–0.97**) | 0.94  (0.81–1.08) |
| 7–13 days | 0.86  (**0.83–0.89**) | 0.88  (**0.85–0.91**) | 0.86  (0.73–1.02) | 0.86  (**0.84–0.89**) | 0.88  (**0.85–0.91**) | 0.83  (**0.73–0.95**) |
| ≥14 days | 0.62  (**0.60–0.64**) | 0.65  (**0.62–0.67**) | 0.69  (**0.58–0.82**) | 0.61  (**0.59–0.63**) | 0.64  (**0.62–0.66**) | 0.73  (**0.64–0.84**) |

Data are adjusted^c^ ORs (95% CIs). **Bold** indicates 95% CIs not crossing 1.0 (significant at p <0.05 level). Child numbers varied according to whether the model analyzed 3, 4, or 5 doses. For child numbers in each group, please refer to Table 2

*CI* confidence interval, *LOS* length of stay, *NICU* neonatal intensive care unit, *OR* odds ratio

^a^Series compliance: age compliant for dose and all previous doses

^b^American Indian or Alaska Native, Native Hawaiian or Other Pacific Islands, or two or more races

^c^ORs were adjusted for birth year, gender, race/ethnicity, NICU hospital stay, birth hospitalization LOS, and basis of Medicaid eligibility
